# “Urological age” as a proxy of healthy longevity: analysis of prospective population-based cohorts in U.S. and China

**DOI:** 10.1097/JS9.0000000000001965

**Published:** 2024-07-25

**Authors:** Yuming Jin, Weichao Huang, Bin Zeng, Lu Yang, Shengfeng Wang, Colucci Manuel, Robesti Daniele, Linghui Deng, Siqi Leng, Dan Hu, Daming Wang, Zhongyuan Jiang, Qinling Yi, Li Zhang, Yuxiao Zeng, Wenjie Zhu, Sheng Li, Xinyue Liu, Qiang Wei, Shi Qiu

**Affiliations:** a Department of Urology, Institute of Urology and National Clinical Research Center for Geriatrics, West China Hospital, Sichuan University; b National Clinical Research Center of Geriatrics, The Center of Gerontology and Geriatrics, West China Hospital, Sichuan University; c West China Biomedical Big Data Center, West China Hospital, Sichuan University; d Department of Clinical Research, West China Hospital, Sichuan University; e Sleep Medicine Center, Mental Health Center, West China Hospital, Sichuan University, Chengdu; f Department of Epidemiology and Biostatistics, School of Public Health, Peking University, Beijing, China; g Institute of Oncology Research (IOR), Oncology Institute of Southern Switzerland (IOSI), Bellinzona; h Università della Svizzera Italiana; i Laboratories for Translational Research, Neurocenter of Southern Switzerland, Ente Ospedaliero Cantonale, Lugano, Switzerland

**Keywords:** lower urinary tract symptoms, urinary incontinence, urological age, urological dysfunction

## Abstract

**Background:**

Assessing urinary symptoms poses a complex challenge for primary care practitioners. In evaluating urological function, authors’ approach involves constructing an urological age through the analysis of laboratory parameters and indicators of the urinary system.

**Methods:**

Based on the National Health and Nutrition Examination Survey (NHANES), urological laboratory tests and age-related symptoms were included in the development of urological age (UA) and urological age acceleration (UAA) through the Klemera Doubal method. In relation to mortality associated with UAA, the metric was categorized into grades (0, 1, 2) as a discrete variable. The authors investigated the correlation between UAA and its grades with mortality, conducted survival analysis based on UAA grades, and explored the correlation between multi-system ageing-related disorders and UAA grades based on the NHANES and the West China Natural Population Cohort Study.

**Results:**

UA was related to age with the r to 0.85 in men and 0.84 in women. Each year the increase in UAA was related to higher 1% and 4% mortality for men and women. Those with UAA grades 1 and 2 were associated with more risk of mortality than individuals with UAA grade 0 (men 8% and 40%, women 24% and 157%). The advanced UAA grades kept pace with multi-system ageing. Healthy diets and lifestyle habits are associated with lower UAA.

**Conclusion:**

Urological age is related to multi-system ageing and increases mortality risk, and urological age can be used to screen high-risk individuals and inform precision clinical development for ageing intervention.

## Background

Highlights

- The determination of urological age, derived from laboratory analyses and symptomatology, exhibits prognostic utility in predicting survival outcomes and demonstrates a correlative relationship with the manifestation of diverse adverse events.
- The validity of the correlation between urological age and adverse outcomes has been substantiated across populations in both the United States and China.
- The urological age can serve as a useful tool in identifying populations at risk of adverse outcomes stemming from diminished urinary system function.

According to demographic projections, by the year 2050, the proportion of the global population aged 65 and above is expected to nearly double compared to the present^1^. This phenomenon is attributed to advancements in social welfare and health care^2,3^. However, amidst the extended lifespan, the emphasis on maintaining a high quality of life should not be underestimated. Progressive decline in urinary function with age continuously diminishes the quality of life for older adults^4,5^. Nevertheless, a considerable number of individuals experiencing urinary functional decline only seek medical assistance when the condition becomes intolerable or severely impacts their quality of life^6–8^. Owing to apprehensions about potential discomfort during medical consultations and a limited understanding of the risks associated with urinary system dysfunction, older adults frequently tolerate urological symptoms without actively seeking medical intervention^9^. This hesitation is often influenced by feelings of shame or the misconception that such changes are a normal part of the ageing process, undoubtedly curtailing the span of a healthy life^7,8^.

The shortened health lifespan resulting from urinary functional decline requires more meticulous screening, whereas current urinary function assessments predominantly focus on individual organs. Common parameters in renal function evaluation include endogenous creatinine clearance rate, serum creatinine, blood urea nitrogen, and blood uric acid are commonly used^10^. Voiding diary requires patients to record urination, leakage, and urination to assess bladder function^11^. Besides, the International Prostate Symptom Score (IPSS) can greatly evaluate the symptoms of benign prostatic hyperplasia^12^. However, the limitation of relying on single-organ assessments becomes apparent, as it overlooks the broader urinary system function, often impacted by age-related urological dysfunction. Biological age (BA) emerges as a method to evaluate an individual’s physiological function, offering a more comprehensive evaluation of biological function and better prediction of adverse outcomes when compared to chronological age^13–15^. Building upon Klemera Doubal (KD) methods, we developed urological age (UA) and urological age acceleration (UAA) to establish a biological age assessment model from the urinary system’s perspective.

Based on the predictive capacity of BA for adverse events, our objective is to leverage laboratory indicators related to the urinary system and urinary system symptoms within the National Health and Nutrition Examination Survey (NHANES) dataset to calculate UA and UAA. Utilizing follow-up mortality data, we aimed to predict the prognostic implications of UA and UAA on mortality outcomes and turn UAA into a categorical variable to distinguish high-risk individuals, thereby facilitating the precise identification of individuals with urological dysfunction who are notably susceptible to elevated risk of mortality. Additionally, data from the West China Natural Population Cohort Study (WCNPCS) will be used by us to verify the relevant results of UA.

## Methods

**Figure 1:**
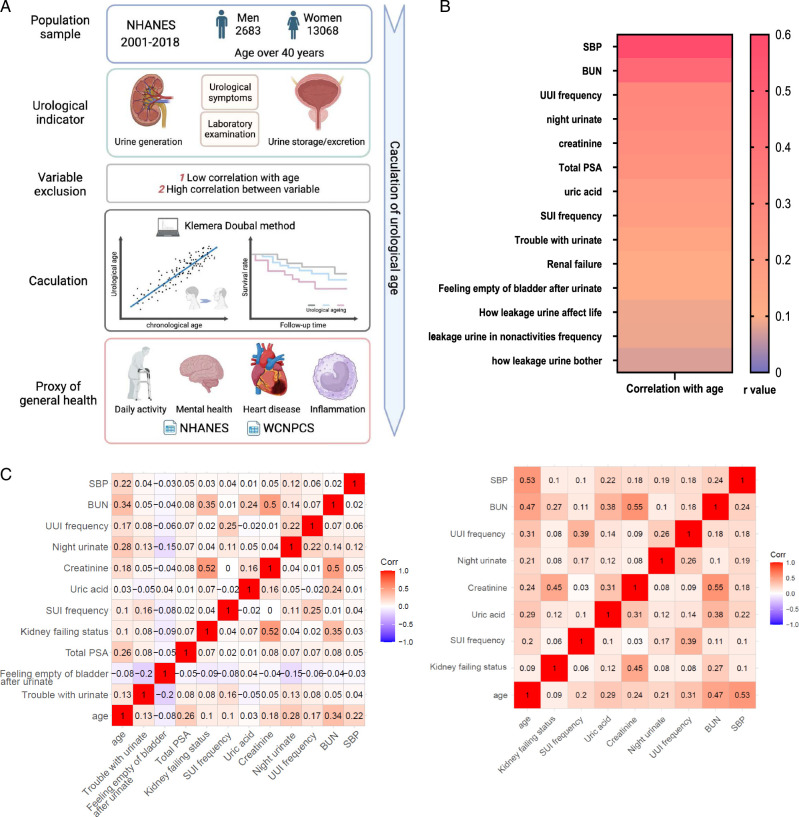
Urological age model and development. (A) The construction and validation of urological age. The icons used in the figures of this study were obtained from BioRender.com. (B) The correlation between urological indicators and age. (C) The association between each urological indicator. (D) Calculate the equation for urological age. NHANES is the National Health and Nutrition Examination Survey. WCNPCS is the West China Natural Population Cohort Study. *x* is the measured value of the biomarker. For each biomarker i, the parameters k, q, and s are derived from regression estimates of the calendar age of the biomarkers in the reference sample. *k*, *q*, and *s* are the regression intercept, slope, and root mean square error, respectively. The *s*_*KD*_ is a scaling factor equal to the square root of the variance of calendar age explained by the biomarker set in the reference sample. *CA* is the chronological age. BUN, blood urea nitrogen; PSA, prostate-specific antigen; SBP, systolic blood pressure; SUI, stress urinary incontinence; UUI, urge urinary incontinence.

The methods of calculating UA in this study are shown in Figure 1A.

We involved urological test examination and symptoms from the NHANES, and we excluded variables with lower age correlation and variables with higher collinearity. UA was calculated through KD methods based on the urological test examination and symptoms after exclusion.

### Participants

The NHANES is conducted by the Centers for Disease Control and Prevention (CDC) and the National Center for Health Statistics (NCHS). The NHANES employs a sophisticated, stratified, multi-stage probability sampling framework to gather data from roughly 5000 individuals on an annual basis^16^. The NHANES protocol has received approval from the Research Ethics Review Board of the National Center for Health Statistics, and all study participants have finished written informed consent. Between the years 2003 and 31 December 2015, mortality data relied upon linked information supplied by the Centers for Disease Control and Prevention (CDC). We merged the NHANES database and the following data. The all-cause mortality was defined based on the International Classification of Diseases, 10th revision (ICD-10), and was assessed by the National Death Index (National Center for Health Statistics).

The WCNPCS, conducted between May 2019 and June 2021, sourced its data from Sichuan Province in Western China. Details about the study were provided in a previous study^17^. All methods followed the principles of the Helsinki Declaration, and informed consents were obtained from participants prior to the study.

The inclusion and exclusion criteria for the population are described in the supplementary document.

### Urological variables including

We included urological symptoms from NHANES as follows:

Urine generation: renal failure status, blood urea nitrogen (BUN), blood creatinine, uric acid, and systolic blood pressure (SBP).

Urine storage and elimination: urge urinary incontinence (UUI) frequency, stress urinary incontinence (SUI) frequency, night urinate times, trouble with urinating, feeling empty of bladder after urinating, how urinary leakage affects life, urinary leakage in non-activities frequency, and how urine leakage bother, and total prostate-specific antigen (PSA).

To calculate urological age, we excluded variables related to age with r less than 0.1. To eliminate the mutual influence between variables, the relations between each two variables were evaluated and those variables with r more than 0.7 would be further considered. The definition of each urological variable is shown in Table S1, Supplemental Digital Content 2, http://links.lww.com/JS9/D182.

### Calculation of urological age, urological age acceleration

The measurement of UA was based on the KD method^18^. The KD method is based on a series of regressions of individual biomarkers on chronological age in a population, obtaining information from chronological regression lines regressed on biomarkers. According to KD methods, the UA represents the level of urinary function of this individual among the population in terms of age. To mathematically distinguish between non-pathological urinary dysfunction and unhealthy urinary dysfunction that occurs during the ageing process, we determined UAA as UA minus age. Consequently, the lower UAA suggested that the individual keeps urological function with a tendency towards younger, and the higher UAA indicated the individual’s urological function with a tendency towards older. To enhance the identification of mortality outcomes associated with elevated UAA, UAA was categorized into grades (0, 1, 2) as a discrete variable, employing a tool for outcome-based cut-point optimization^19^. The higher grades in UAA indicate a decline in the function of the urinary system.

### UAA as a proxy of general health

To assess whether the UAA above grades could identify the condition of general health deterioration, we investigated the following domains in NHANES:

Daily activity: Activities of daily living (ADL) and instrumental activities of daily living (IADL) revealed the day-living ability of the individual^20^. Mental health: The Patient Health Questionnaire (PHQ) is a depression symptom-scoring questionnaire, a higher score of PHQ suggests worsening depression symptoms^21^. Cardiovascular morbidity: Cardiometabolic index (CMI) has significance in the screening of diabetes, atherosclerosis, and renal dysfunction. The occurrence of cardiovascular diseases was defined by the report of participants^22^. Systemic inflammation: The neutrophil-to-lymphocyte ratio (NLR) and C-reactive protein (CRP) are significant inflammatory markers^23^. Additionally, UA based on the WCNPCS was conducted by using similar urinary system indicators as in NHANES. The association between age and UA, and the proxy of general health of UAA were evaluated based on the WCNPCS.

### Statistical methods

IBM SPSS 26.0 software and the statistical software packages R (http://www.R-project.org, The R Foundation) were used in the statistical analyses. In our study, continuous variables were submitted as the mean ± standard deviation (SD), and categorical variables as proportions. We conducted linear regression to evaluate the association between urological variables and age. The relation between all‐cause mortality with UA and UAA was assessed by the Cox regression. The probability of survival of men and women using the Kaplan–Meier method and log rank was used in comparing the survival differences among participants in UAA grades. Adjusted Cox regression model to evaluate the association between UAA grades and all‐cause mortality, and the model adjusted for the sociodemographic variables and biological variables. The association between UAA grades and dysfunction of multiple systems was assessed by the adjusted linear and logics regression. Two-tailed *p* values were performed with a significance level of less than 0.05. When analyzing NHANES data, MEC weights were used to adjust and obtain representative data. This work has been reported in line with the STROCSS criteria, Supplemental Digital Content 1, http://links.lww.com/JS9/D181 ^24^.

## Results

### Urological variable selection and participants characteristics

Based on the urological variables mentioned before, we excluded three variables for the low correlation with age: how leakage urine affects life, leakage urine in non-activities frequency, and how urine leakage bothers (shown in Fig. 1B). No variable was excluded for the high correlation between each other (shown in Fig. 1C). We included SBP, BUN, UUI frequency, night urinate, creatinine, uric acid, SUI frequency, renal failing status, total PSA, feeling empty of the bladder after urinating and trouble with urination, to calculate urological age for men. Note that for the women cohort, the parameters of feeling empty of bladder after urinating, total PSA and trouble with urination were excluded. The calculation of UA and UAA is shown in Figure S1, Supplemental Digital Content 2, http://links.lww.com/JS9/D182.

According to the urological variable involved in the calculation of urological age, we included 2683 men and 13 068 women over 40 years from NHANES 2001–2018. The mean age was 59.56 for men and 59.67 for women. Among the participants, SUI and UUI were more frequently observed in women (48.0% and 37.5%, respectively) than in men (SUI 4.8%, UUI 18.9%). The baseline characteristics of the study participants in NHANES are depicted in Table S2, Supplemental Digital Content 2, http://links.lww.com/JS9/D182.

### All‐cause mortality and survival risk increased along with UA increased

**Figure 2:**
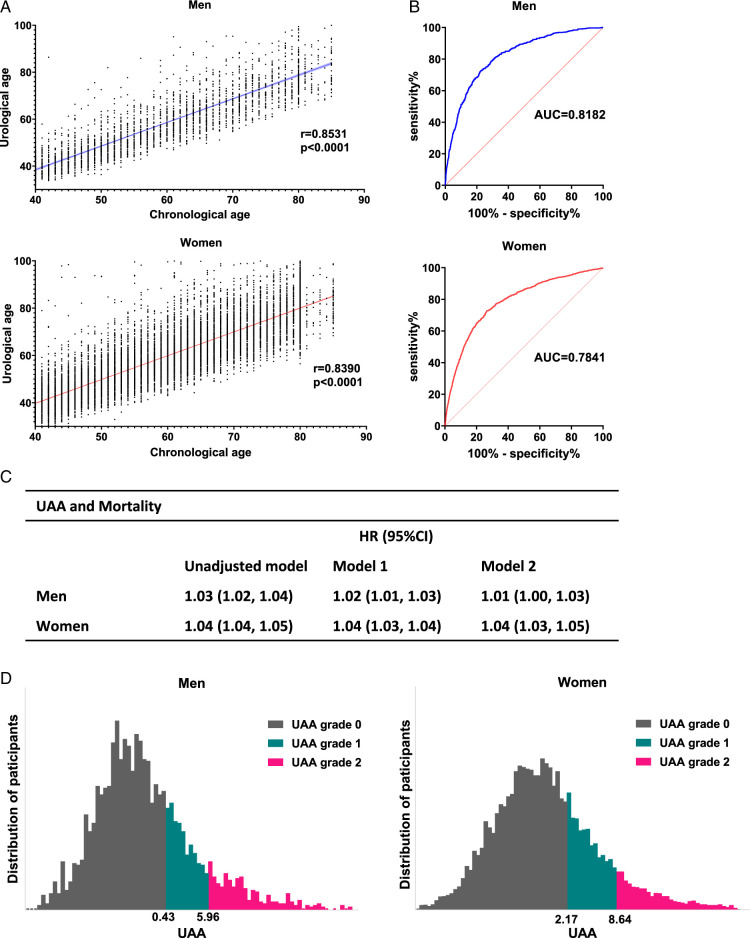
Urological age, UAA, and UAA grades. (A) The correlation between urological age and age among men and women. (B) Receiver operating characteristic curve about the prediction of UA on mortality. (C) The association between UAA and mortality. Model 1: adjusted for age, race, education and poverty income ratio. Model 2 adjusted for age, race, education, poverty income ratio, BMI, drinking and smoking. (D) The distribution of UAA in participants and the cut-off points of UAA for UAA grades, which were evaluated by X-tile. AUC, area under the curve; HR, hazard ratio; UAA, urological age acceleration.

We first evaluated the prediction of UA on mortality and subsequently calculated UAA grades according to the correlation with mortality. UA was associated with age among men and women with r=0.85 and r=0.82 (shown in Fig. 2A). The area under the curve (AUC) was 0.82 and 0.78 for men and women, respectively (Fig. 2B). The correlation between UAA and all-cause mortality illustrates that for every 1-year increase in UAA, adjusted all-cause mortality rises by 1% and 4% among men and women, respectively (Fig. 2C). Regarding the association between UAA and survival, the identified cut-off points for UAA grades were 0.43 and 5.96 for men, and 2.17 and 8.64 for women (Fig. 2D). Detailed baseline information for participants categorized by UAA grades is presented in Table S3, Supplemental Digital Content 2, http://links.lww.com/JS9/D182 for men and Table S4, Supplemental Digital Content 2, http://links.lww.com/JS9/D182 for women.

**Figure 3:**
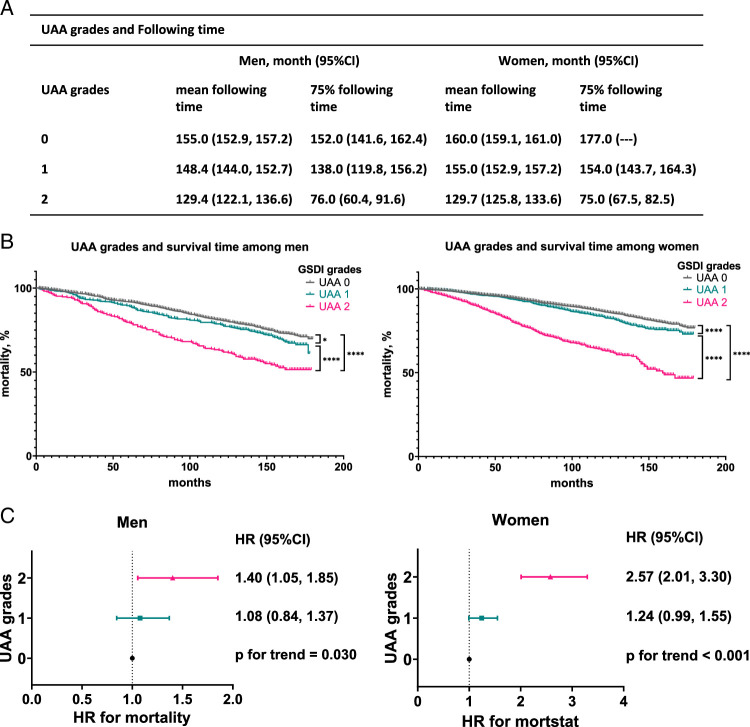
Survival analysis based on the UAA grades. (A) UAA grades and following time. (B). Survival analysis based on the UAA grades among men and women. (C) The association between UAA grades and all-cause mortality. HR, hazard ratio; UAA, urological age acceleration.

To gain clinical insight into UA-associated changes, we analyzed the association between UAA grades and all‐cause mortality. Survival analysis suggested that with an increase in UAA grade, the survival time decreased among men and women (submitted in Fig. 3A). In crude Kaplan–Meier analyses, significant differences were observed between UAA groups among men and women, through three pairwise comparisons in sex (shown in Fig. 3B). Additionally, with the increase in UAA grade, the risk of mortality was also increasing (Fig. 3C).

### Advanced UAA and prevalence of ageing-related disorders

Given that ageing-related disorders were observed at multi-system levels, we performed multiple linear regression and multivariate logistic regression to investigate the association between UAA grades and deterioration of the ability of daily living, mental health, cardiovascular system, nutritional status, and systemic inflammation. Overall, these results suggest that the UAA may be considered as a proxy of general health in both men and women.

**Figure 4:**
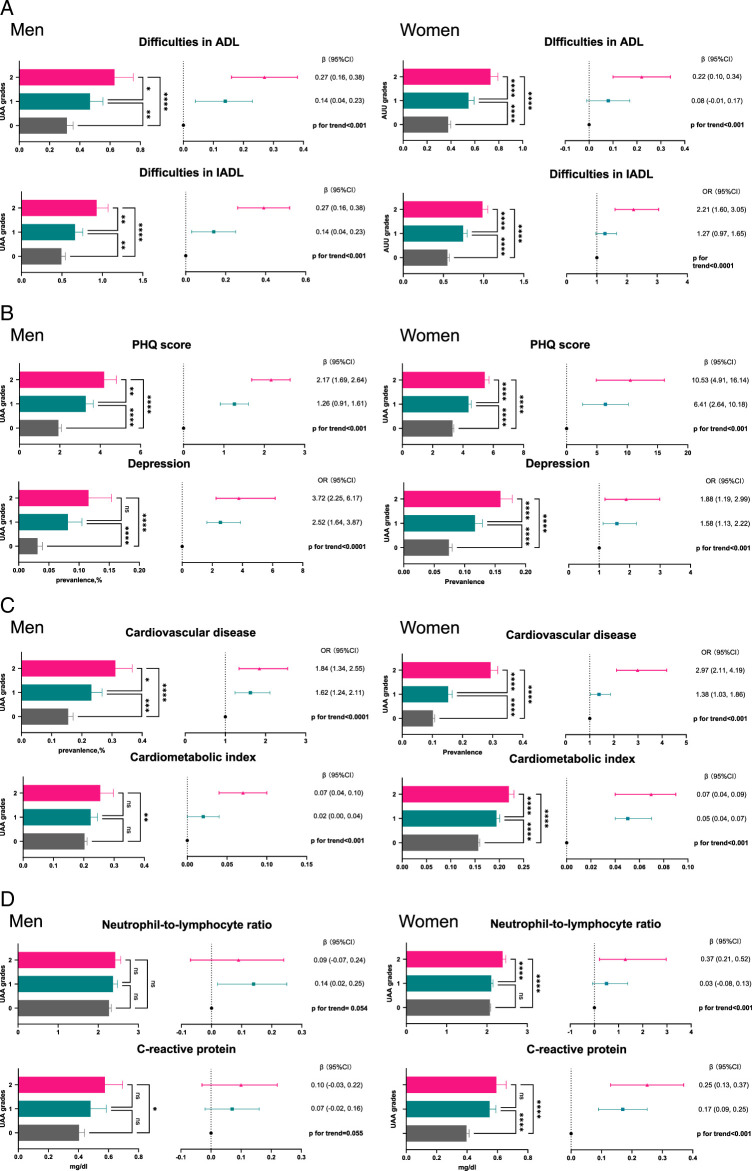
The association between UAA grades and multi-system ageing among men and women. The association between ageing-related disorders and UAA grades. (A) Activities of daily living (ADL) and instrumental activities of daily living (IADL). (B) The Patient Health Questionnaire (PHQ) score and depression. (C) Cardiometabolic index and cardiovascular diseases. (D) Neutrophil-to-lymphocyte ratio and C-reactive protein. The association adjusted for age, race, education, poverty income ratio, BMI, drinking, smoking, met. UAA, urological age acceleration.

With the increasing UAA grade, participants faced more difficulties in ADL and IADL, in men and women. As UAA grade increases, the trend of more difficulties in ADL/IADL was significant both in men and women. Depression was defined as a score of PHQ greater than 10. We observed a trend of increasing grades of UAA companies with higher scores of PHQ and higher prevalence of depression, which indicated those with higher grades of UAA lived with poor mental health (Fig. 4B). There was a significant positive correlation between UAA grades and CMI among men and women. A higher prevalence of cardiovascular disease was observed among those with UAA grades 2 and 1 than those with UAA grades 0 among men and women(Fig. 4C). Higher UAA grades were related to a higher level of CRP and NLR with a nearly significant trend among men (Fig. 4D). Additionally, higher UAA grades were associated with higher random blood glucose levels in both men and women (Figure S2, Supplemental Digital Content 2, http://links.lww.com/JS9/D182). In women, higher UAA grades were also associated with malnutrition and higher concentrations of klotho protein (Figure S2, Supplemental Digital Content 2, http://links.lww.com/JS9/D182).

### UA and UAA in west China

**Figure 5:**
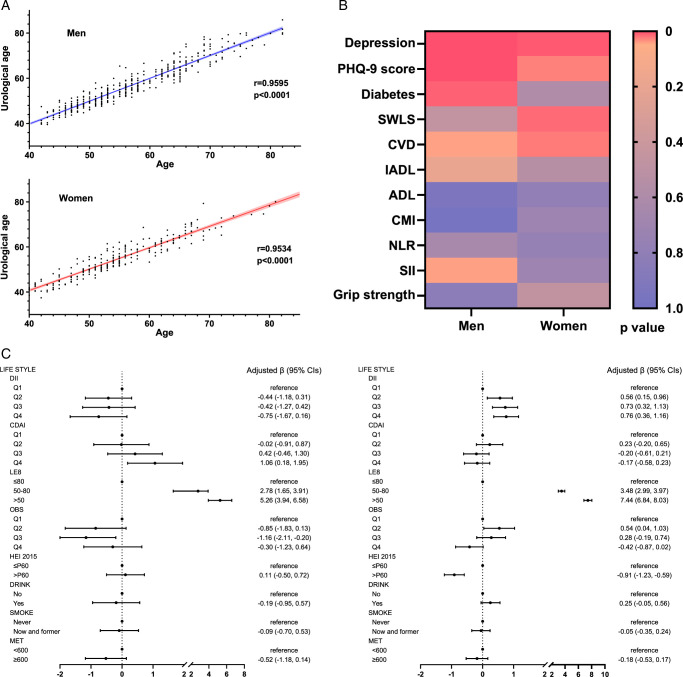
Urological age and health correlations in WCNPCS, and the association between lifestyles and urological age acceleration (UAA) in NHANES. (A) The association between urological age and age is based on WCNPCS. (B) The association between urological age acceleration and health indicator. (C) The association between lifestyles and UAA in NHANES. The associations were adjusted for age, race, education, poverty income ratio, and BMI. ADL, difficulties in activities of daily living; CDAI, composite dietary antioxidant index; CMI, the cardiometabolic index; CVD, cardiovascular diseases; DII, Dietary Inflammatory Index; IADL, difficulties in instrumental activities of daily living; LE8, Life’s Essential 8; MET, the metabolic equivalent; NLR, the neutrophil-to-lymphocyte ratio; OBS, oxidative balance score; PHQ, Patient Health Questionnaire; SII, systemic immune-inflammation index; SWLS, Scale to Measure Satisfaction with Life.

Utilizing analogous urological variables, we formulated the UA in all ages and observed a pronounced correlation with age (r =0.95 in men and r =0.95 in women, Fig. 5A). Furthermore, empirical evidence from Chinese datasets substantiates the association between UA levels and conditions such as depression, diabetes, cardiovascular disease, and the systemic inflammation index (Fig. 5B). Additionally, in women, higher UAA is associated with lower life satisfaction. The correlation between age and urological indicator based on WCNPCS is shown in Table S5, Supplemental Digital Content 2, http://links.lww.com/JS9/D182. The baseline characteristics of the study participants in NHANES are depicted in Table S6, Supplemental Digital Content 2, http://links.lww.com/JS9/D182.

### UAA and lifestyles

Using NHANES data, we supplemented our analysis with various interventions and their associations with UA/UAA. We found that in women, a higher Dietary Inflammatory Index (DII), a higher oxidative balance score (OBS), and a lower HEI-2015, and in men, a higher Composite Dietary Antioxidant Index (CDAI), as well as a lower Life’s Essential 8 (LE8) in the overall population, are all associated with higher UAA. This suggests that healthy diets and lifestyle habits can mitigate the increase in UAA and slow down urinary system ageing (Fig. 5C).

## Discussion

UA and UAA can serve as effective tools for evaluating health life span. This approach is worth considering alongside other assessment indicators related to urinary function and symptoms. Due to the critical role of kidney function in maintaining internal balance, kidney dysfunction has been demonstrated to be associated with an increased risk of mortality^25^. Reduced kidney function is associated with an increased risk of cardiovascular disease-related mortality^26^. However, no significant association has been found between reduced kidney function and the risk of cardiovascular events or overall mortality^26,27^. IPSS can also be utilized to assess the severity of lower urinary tract symptoms (LUTS) in women^28^. Previous studies have assessed the potential association of the IPSS with depression, possibly mediated through factors such as sleep disturbances and the presence of multiple medical conditions^29^. However, the association between the IPSS and the risk of mortality has not been elucidated. In this study, we observed a concurrent increase in the UAA grades and a decline in multi-system functionality. For the study design, we cannot conclude that urinary system dysfunction directly causes multi-system disorders in the body. The elevation in UAA grades not only helps identify urinary dysfunction associated with increased mortality risk but also serves as an indicator for potential abnormalities in other system functions.

This work opens new perspectives by introducing a comprehensive approach to assessing urinary symptoms and urological function. The integration of urinary system laboratory tests and symptoms to calculate the overall biological age of the urinary system, as represented by UAA, provides a novel and nuanced method for evaluating mortality risk. The concurrent increase in UAA grades and decline in multi-system functionality raises intriguing questions about potential interactions between urinary dysfunction and broader systemic health, paving the way for future research to explore these complex relationships and their implications for clinical practice. Overall, this work broadens the perspective on the significance of UAA and urinary assessments in understanding mortality risk and health life span.

This investigation has certain constraints that need to be discussed. Firstly, our study exclusively enrolled individuals aged 40 and above, potentially constraining the generalizability of UAA within the context of this study. The inclusion of prostate symptom questionnaires for men aged 40 and older in the NHANES dataset influenced our study’s age range criteria for women accordingly. This selection criterion may overlook individuals experiencing urinary function decline earlier in their lifespan. In the NHANES database, UA/UAA remains significantly associated with mortality and multi-system ageing in women under 40 (Supplementary Figure S3, Supplemental Digital Content 2, http://links.lww.com/JS9/D182). Additionally, we constructed UA/UAA across all age groups in the West China Natural Population Cohort, showing a correlation between UAA and multi-system health. Given that prostate symptoms increase with age, it is possible to construct UA/UAA consistent with age in men under 40. However, whether UA/UAA is associated with survival in men under 40 requires further evidence. Secondly, since the relationship between UAA and multi-system health is based on cross-sectional studies, we cannot establish a causal relationship between UA/UAA and multi-system health. We used multiple population samples to validate the effectiveness of UA, but it cannot be concluded that an elevation in the UAA directly precipitates an increased risk of multi-system functional decline. Thirdly, the selection of the NHANES and natural population databases may introduce selection bias. This study included data from only two countries. Other publicly available datasets do not have sufficient urinary system symptom data to construct UA/UAA, making this bias unavoidable. To minimize bias, NHANES data were sourced from the United States, the world’s largest economy, while the West China Natural Population Cohort data were sourced from China, the largest developing country. This enhances the representativeness of our study. Furthermore, both studies are based on population data rather than samples collected from hospitals or care institutions, and they feature high questionnaire response rates. Fourthly, the association between UA/UAA and health has only been validated in the US and China. Although data from these countries are somewhat representative, as mentioned earlier, further validation is needed with data from other regions to confirm its applicability. Fifthly, the construction of UA/UAA is based on the subjective responses of participants, which inevitably introduces recall bias. However, in both cohorts, the questionnaires were administered by trained professionals who assisted participants in providing accurate responses. Given the subjective nature of urinary system symptoms, subjective reporting can be considered a valid standard for disease assessment^30,31^, responses to symptoms can still, to a certain extent, represent the patient’s urinary system health.

## Conclusion

UA and UAA were derived from laboratory test results and symptoms related to the urinary system. Elevations in both UA and UAA levels are correlated with an augmented risk of mortality. Moreover, higher UAA grades exhibit a connection with multi-system functional decline. This underscores the utility of UA in offering insights into broader health life span decline associated with urinary ageing. A healthy lifestyle is associated with lower UAA.

## Ethical approval

This study was registered with the Chinese Clinical Trial Registry (No. ChiCTR1900024623) and received ethical approval from the committee at West China Hospital of Sichuan University on 1 March 2022. Informed consents were obtained from participants prior to study. https://www.chictr.org.cn/showproj.html?proj=40590. And the WCNPCS has already published research in other fields with doi:10.1186/s12889-022-14187-5.

## Consent

Written informed consent was obtained from the patient for publication of this case report and accompanying images. A copy of the written consent is available for review by the Editor-in-Chief of this journal on request.

## Source of funding

This work was supported by the National key research and development program of China (2022YFC3602902, 2022YFC3602901, 2020YFC2008600), Programs from Science and Technology Department of Sichuan Province (2023NSFSC1906, 2021YJ0462), National Clinical Research Center for Geriatrics, West China Hospital, Sichuan University (Z2023LC007).

## Author contribution

Y.J., S.Q., D.H. and Z.Z. designed this study. L.Y., W.H. and W.Z. supervised the study. S.Q., Z.J., Q.Y. and L.Z. collected and provided the data. C.M., R.D. and S.W. accessed and verified the data. Y.J., S.L. and Y.Z. Analyzed the data and interpreted the results. B.Z., X.L. and X.Z. wrote the original draft. Q.W., L.D. and L.S. revised the manuscript. Q.W., D.W., L.S. and X.L. provided administrative, technical, or material support. All authors had full access to all the data in the study and had final responsibility for the decision to submit for publication.

## Conflicts of interest disclosure

The authors have no conflicts of interests to disclose.

## Research registration unique identifying number (UIN)

This study was registered with the Chinese Clinical Trial Registry (No. ChiCTR1900024623). https://www.chictr.org.cn/showproj.html?proj=40590.

## Guarantor

Shi Qiu and Qiang Wei.

## Data availability statement

The NHANES datasets used for these analyses are publicly available at: https://wwwn.cdc.gov/nchs/nhanes/default.aspx. S.Q. had full access to all the data in the WCNPCS study and takes responsibility for the integrity of the data and the accuracy of the data analysis.

## Provenance and peer review

Not applicable.

## Supplementary Material
