# Supplementary material for: “Urological age” as a proxy of healthy longevity: analysis of prospective population-based cohorts in U.S. and China
Source: Int J Surg. 2024 Jul 25;111(1):502–11. doi: 10.1097/JS9.0000000000001965 (PMC11753421; doi:10.1097/JS9.0000000000001965)
Supplement: Supplementary file 2 [file js9-111-0502-s002.docx]

1. Evaluation of multisystem senescence and covariables

ADL and IADL

Activities of daily living (ADL) indicate the function of daily living, including getting in and out of bed, using tools to eat, dressing, etc(1). Instrumental activities of daily living revealed the ability of the individual's ability to use daily tools, such as cooking food, financial management, household chores, etc (1). In our study, ADL consisted of getting in and out of bed, using tools to eat, and dressing. IADL consisted of cooking food, financial management, and household chores.

PHQ and depression

The depression screening questionnaire employed in NHANES comprises nine items sourced from the Patient Health Questionnaire (PHQ-9)(2). All participants within the Mobile Examination Center (MEC) were eligible to undergo this depression screener. The PHQ-9, a self-report instrument, is a well-validated and reliable assessment tool consisting of nine items that align with the diagnostic criteria for a major depressive episode (3).

CMI and CVD

Cardiac metabolic index, a new metabolic index was composed of clinical indicators of high-density lipoprotein cholesterol (HDL single bond C), triglyceride (TG) and waist-height ratio (WHtR)(4). WHtR is mainly used to measure the degree of obesity, and TG/HDL-C is used to evaluate the level of blood lipids. The CMI showed that CMI could well reflect the degree of obesity and the level of blood lipids, and it was an independent risk factor for the occurrence of diabetes.

Cardiovascular disease (CVD) was diagnosed using self-reported physician diagnoses obtained during an individual interview using a standardized medical condition questionnaire. The participants were asked, “Has a doctor or other health expert ever informed you that you have CHF/CHD/angina pectoris/MI/stroke?” A person was regarded as having CVD if he or she replied “yes” to any of the above questions. The result was transformed to a binary variable.

NLR and CRP

neutrophil-to-lymphocyte ratio (NLR) was positively associated with the prevalence of hypertension(5). NLR = neutrophils/lymphocytes. C-reactive protein (CRP) is a nonspecific marker of inflammation. Assays were performed on a Behring Nephelometer to determine quantitative CRP levels.

CONUT

CONtrolling NUTritional status (CONUT) is an efficient tool for early detection and continuous control of hospital under nutrition, with the suitable characteristics for these screening functions. The calculation of CONUT was described in previous study(6).

Klotho

Alpha-Klotho, also commonly referred to simply as Klotho, is a multifunctional protein, describing a gene mutation in mice involved in aging and arteriosclerosis(7). Klotho levels were measured in individuals who were between the ages of 40 and 79 years, had pristine serum samples available, and had consented to the use of their samples for future research in NHANES.

Physical activity was assessed by metabolic equivalent scores (MET) = vigorous work-related activity*8 + moderate work-related activity *4 + walking or bicycling for transportation*4 + vigorous leisure-time physical activity*8 + moderate leisure-time physical activity*4.

Income-to-poverty ratio (family income divided by federal poverty threshold) provided a measure of socio-economic status and was analyzed by quartiles(8).

The Composite Dietary Antioxidant Index (CDAI) serves as a dependable and accurate nutritional instrument for evaluating the overall antioxidant properties of a diet. It is a cumulative score that encompasses six dietary antioxidants: vitamins A, C, and E, as well as manganese, selenium, and zinc(9).

The primary objective in developing the original Dietary Inflammatory Index (DII) in 2009 was to create a tool capable of classifying individuals' diets along a spectrum ranging from highly anti-inflammatory to highly pro-inflammatory(10).

The Healthy Eating Index (HEI)-2015 is an extensive measure of an individual's dietary habits, providing both overall and subcomponent scores, in alignment with the 2010 Dietary Guidelines for Americans(11).

Life’s Essential 8 (LE8) includes significant updates, such as the addition of sleep quality indicators and enhanced scoring algorithms, improving upon the original Life’s Simple 7(12). The components of Life's Essential 8 encompass an updated diet, physical activity, revised nicotine exposure, newly added sleep health, body mass index, updated blood lipids, modified blood glucose, and blood pressure.

The oxidative balance score (OBS) was developed to quantify individual exposure to pro-oxidants and overall antioxidants. A higher OBS signifies a predominance of antioxidants over pro-oxidants(13).

The Satisfaction With Life Scale (SWLS) is specifically designed to measure overall life satisfaction and does not address related constructs such as positive affect or loneliness. It has demonstrated favourable psychometric characteristics, including high internal consistency and strong temporal reliability(14).

2. The calculation of UA based on the West China Natural Population Cohort Study(WCNPCS)

We included UUI(yes, no), SUI(yes,no), night urinate, SBP, blood creatine, bun to calculated the UA for women, and added trouble with urinating, feeling empty of bladder after urinating for men. The correlation between age and urological indicator was shown in Table S5. The correlation between each urological indicator was shown in Figure S1. We involved 412 men and 244 women with full data in our study, with means age 56.4 and 53.9, respectively. The baseline of characters of participants was shown in Table S6.

3.Supplement of statistical methods

Logistic and linear regression to evaluate the association between urological variables and age, and no variable was adjusted in the regression model.

The Cox proportional hazards regression was performed to evaluate the relation between all‐cause mortality with UA and UAA, we adjusted for age, race, education, income-to-poverty ratio, MET, BMI, smoking, drinking.

The association between UAA grades and dysfunction of multiple systems was assessed by the adjusted linear and logics regression, and age, race, education and income-to-poverty ratio were adjusted in the model.

4. Inclusion and Exclusion Criteria of the Research Sample

Due to changes in the collection of urinary system variables in NHANES, we utilized samples with complete relevant indicators from 2001 to 2020 to explore the correlation between age, urinary symptoms, and laboratory indicators. For constructing the UAA/UAA index and investigating its association with mortality and multisystem ageing, we included female populations from 2005 to 2020 and male populations (aged 40 and above) from 2005 to 2008. Subsequently, we validated the correlation between UA/UAA and mortality and multisystem ageing in females under 40 years old, including the female population from NHANES from 2005 to 2020. When validating the correlation between UAA and multisystem ageing using the West China Natural Population Cohort Study (WCNPCS), we included populations of all ages that met the criteria for UA calculation.
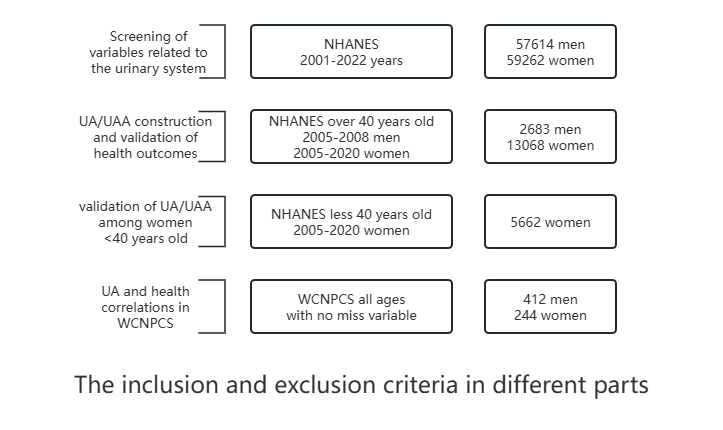


Table S1. The definition of each urological variable.

| Variable | Unit/categories |
| --- | --- |
| BUN | mg/dL |
| Serum creatinine | mg/dL |
| Serum uric acid | mg/dL |
| Total PSA | ng/mL |
| Systolic blood pressure | mmHg |
| Renal failure status | No,  yes but no dialysis  yes and dialysis |
| SUI frequency | No  Less than once a month,  A few times a month,  A few times a week  Every day or every night |
| UUI frequency | No  Less than once a month  A few times a month  A few times a week  Every day or every night |
| Night urinate, time | 0,1,2,3,4,5 or more |
| Trouble with urinating | yes  no |
| Feeling empty of bladder after urinating | yes  no |
| How UI affect life | Not at all  only a little  somewhat  very much  greatly |
| UI in nonactivities frequency | No  Less than once a month,  A few times a month,  A few times a week  Every day or every night |
| how urine leakage bother | Not at all  only a little  somewhat  very much  greatly |

BUN blood urea nitrogen, SBP systolic blood pressure, SUI stress urinary incontinence, UUI Urge urinary incontinence, Total PSA prostate-specific antigen.

Table S2. Baseline characteristics of the study participants classified by sex.

| Characteristics | Total | Gender | | p-value |
| --- | --- | --- | --- | --- |
|  |  | Men | women |  |
| N, count | 15751 | 2683 | 13068 |  |
| Age, year | 59.65 ± 12.20 | 59.56 ± 12.51 | 59.67 ± 12.14 | 0.457 |
| Race, n (%) |  |  |  | 0.013 |
| mexican American | 2270 (14.41%) | 429 (15.99%) | 1841 (14.09%) |  |
| other Hispanic | 1537 (9.76%) | 185 (6.90%) | 1352 (10.35%) |  |
| non-Hispanic White | 7205 (45.74%) | 1487 (55.42%) | 5718 (43.76%) |  |
| non-Hispanic Black | 3350 (21.27%) | 504 (18.78%) | 2846 (21.78%) |  |
| other Race | 1389 (8.82%) | 78 (2.91%) | 1311 (10.03%) |  |
| Education, n (%) |  |  |  | <0.001 |
| less Than 9th Grade | 1878 (11.93%) | 439 (16.36%) | 1439 (11.02%) |  |
| 9-11th Grade | 2105 (13.38%) | 391 (14.57%) | 1714 (13.13%) |  |
| high School Grad/GED or Equivalent | 3710 (23.58%) | 632 (23.56%) | 3078 (23.58%) |  |
| some College or AA degree | 4598 (29.22%) | 640 (23.85%) | 3958 (30.32%) |  |
| college Graduate or above | 3445 (21.89%) | 581 (21.65%) | 2864 (21.94%) |  |
| Poverty income ratio | 2.64 ± 1.62 | 2.83 ± 1.63 | 2.60 ± 1.62 | <0.001 |
| Drinking |  |  |  | <0.001 |
| No | 5061 (32.16%) | 497 (18.54%) | 4564 (34.95%) |  |
| Yes | 10678 (67.84%) | 2183 (81.46%) | 8495 (65.05%) |  |
| Smoking |  |  |  | <0.001 |
| never | 9132 (58.01%) | 1018 (37.96%) | 8114 (62.12%) |  |
| current | 2606 (16.55%) | 604 (22.52%) | 2002 (15.33%) |  |
| quitter | 4005 (25.44%) | 1060 (39.52%) | 2945 (22.55%) |  |
| BMI | 30.03 ± 7.27 | 28.88 ± 5.83 | 30.26 ± 7.51 | <0.001 |
| MET | 3212.34 ± 5263.36 | 3585.62 ± 6082.10 | 3124.92 ± 5048.89 | <0.001 |
| BUN, mg/dL | 14.57 ± 6.37 | 14.82 ± 6.32 | 14.52 ± 6.38 | <0.001 |
| Serum creatinine, mg/dL | 0.87 ± 0.41 | 1.06 ± 0.49 | 0.83 ± 0.38 | <0.001 |
| Serum uric acid, mg/dL | 5.24 ± 1.43 | 6.09 ± 1.36 | 5.07 ± 1.38 | <0.001 |
| SBP, mmHg | 128.11 ± 20.12 | 127.68 ± 18.12 | 128.20 ± 20.51 | 0.97 |
| Renal failure , n (%) |  |  |  | 0.006 |
| no | 15141 (96.13%) | 2603 (97.02%) | 12538 (95.94%) |  |
| yes but no dialysis | 561 (3.56%) | 69 (2.57%) | 492 (3.76%) |  |
| yes and dialysis | 49 (0.31%) | 11 (0.41%) | 38 (0.29%) |  |
| SUI frequency |  |  |  | <0.001 |
| no | 9344 (59.32%) | 2553 (95.15%) | 6791 (51.97%) |  |
| less than once a month | 2711 (17.21%) | 74 (2.76%) | 2637 (20.18%) |  |
| a few times a month | 1978 (12.56%) | 34 (1.27%) | 1944 (14.88%) |  |
| a few times a week | 968 (6.15%) | 14 (0.52%) | 954 (7.30%) |  |
| every day or every night | 750 (4.76%) | 8 (0.30%) | 742 (5.68%) |  |
| UUI frequency |  |  |  | <0.001 |
| no | 10346 (65.68%) | 2177 (81.14%) | 8169 (62.51%) |  |
| less than once a month | 2094 (13.29%) | 257 (9.58%) | 1837 (14.06%) |  |
| a few times a month | 1822 (11.57%) | 165 (6.15%) | 1657 (12.68%) |  |
| a few times a week | 892 (5.66%) | 56 (2.09%) | 836 (6.40%) |  |
| every day or every night | 597 (3.79%) | 28 (1.04%) | 569 (4.35%) |  |
| Night urinate, time |  |  |  | <0.001 |
| 0 | 3709 (23.55%) | 796 (29.67%) | 2913 (22.29%) |  |
| 1 | 5817 (36.93%) | 955 (35.59%) | 4862 (37.21%) |  |
| 2 | 3517 (22.33%) | 565 (21.06%) | 2952 (22.59%) |  |
| 3 | 1757 (11.15%) | 244 (9.09%) | 1513 (11.58%) |  |
| 4 | 570 (3.62%) | 69 (2.57%) | 501 (3.83%) |  |
| 5 or more | 381 (2.42%) | 54 (2.01%) | 327 (2.50%) |  |
| Total PSA, ng/ml |  | 1.85 ± 3.31 |  |  |
| Trouble with urinate |  |  |  |  |
| no |  | 2440 (90.94%) |  |  |
| yes |  | 243 (9.06%) |  |  |
| feeling empty of bladder after urinate |  |  |  |  |
| no |  | 285 (10.62%) |  |  |
| yes |  | 2398 (89.38%) |  |  |

BUN blood urea nitrogen, SBP systolic blood pressure, SUI stress urinary incontinence, UUI Urge urinary incontinence, Total PSA prostate-specific antigen.

The p-value was calculated by the Kruskal-Wallis test for continuous variables and the chi-square test for categorical variables.

Table S3. Baseline characteristics of the men participants classified by UAA grades.

| Characteristics | UAA grades | | | p-value |
| --- | --- | --- | --- | --- |
|  | 0 | 1 | 2 |  |
| N, count | 1869 | 544 | 270 |  |
| Age, year | 59.13 ± 12.37 | 58.85 ± 12.73 | 63.97 ± 12.21 | <0.001 |
| Race, n (%) |  |  |  | 0.004 |
| mexican American | 296 (15.84%) | 91 (16.73%) | 42 (15.56%) |  |
| other Hispanic | 134 (7.17%) | 36 (6.62%) | 15 (5.56%) |  |
| non-Hispanic White | 1072 (57.36%) | 278 (51.10%) | 137 (50.74%) |  |
| non-Hispanic Black | 310 (16.59%) | 124 (22.79%) | 70 (25.93%) |  |
| other Race | 57 (3.05%) | 15 (2.76%) | 6 (2.22%) |  |
| Education, n (%) |  |  |  | <0.001 |
| less Than 9th Grade | 284 (15.20%) | 94 (17.28%) | 61 (22.59%) |  |
| 9-11th Grade | 247 (13.22%) | 88 (16.18%) | 56 (20.74%) |  |
| high School Grad/GED or Equivalent | 443 (23.70%) | 130 (23.90%) | 59 (21.85%) |  |
| some College or AA degree | 464 (24.83%) | 119 (21.88%) | 57 (21.11%) |  |
| college Graduate or above | 431 (23.06%) | 113 (20.77%) | 37 (13.70%) |  |
| Poverty income ratio | 2.94 ± 1.63 | 2.63 ± 1.61 | 2.42 ± 1.57 | <0.001 |
| Drinking |  |  |  | 0.002 |
| No | 314 (16.83%) | 125 (22.98%) | 58 (21.48%) |  |
| Yes | 1552 (83.17%) | 419 (77.02%) | 212 (78.52%) |  |
| Smoking |  |  |  | 0.146 |
| never | 727 (38.92%) | 198 (36.40%) | 93 (34.44%) |  |
| current | 414 (22.16%) | 136 (25.00%) | 54 (20.00%) |  |
| quitter | 727 (38.92%) | 210 (38.60%) | 123 (45.56%) |  |
| BMI | 28.59 ± 5.09 | 29.55 ± 7.54 | 29.52 ± 6.49 | <0.001 |
| MET | 3612.46 ± 5930.00 | 3594.90 ± 6735.09 | 3334.73 ± 5774.64 | 0.103 |
| BUN, mg/dL | 13.70 ± 4.57 | 15.93 ± 6.05 | 20.30 ± 11.77 | <0.001 |
| Serum creatinine, mg/dL | 1.01 ± 0.21 | 1.07 ± 0.26 | 1.42 ± 1.34 | <0.001 |
| Serum uric acid | 6.02 ± 1.28 | 6.20 ± 1.44 | 6.34 ± 1.71 | <0.001 |
| SBP, mmHg | 124.25 ± 15.40 | 132.68 ± 18.55 | 141.42 ± 24.65 | 0.97 |
| Renal failure , n (%) |  |  |  | <0.001 |
| no | 1856 (99.30%) | 515 (94.67%) | 232 (85.93%) |  |
| yes but no dialysis | 13 (0.70%) | 28 (5.15%) | 28 (10.37%) |  |
| yes and dialysis | 0 (0.00%) | 1 (0.18%) | 10 (3.70%) |  |
| SUI frequency |  |  |  | <0.001 |
| no | 1833 (98.07%) | 504 (92.65%) | 216 (80.00%) |  |
| less than once a month | 30 (1.61%) | 26 (4.78%) | 18 (6.67%) |  |
| a few times a month | 6 (0.32%) | 10 (1.84%) | 18 (6.67%) |  |
| a few times a week | 0 (0.00%) | 4 (0.74%) | 10 (3.70%) |  |
| every day or every night | 0 (0.00%) | 0 (0.00%) | 8 (2.96%) |  |
| UUI frequency |  |  |  | <0.001 |
| no | 1691 (90.48%) | 369 (67.83%) | 117 (43.33%) |  |
| less than once a month | 130 (6.96%) | 84 (15.44%) | 43 (15.93%) |  |
| a few times a month | 43 (2.30%) | 65 (11.95%) | 57 (21.11%) |  |
| a few times a week | 3 (0.16%) | 16 (2.94%) | 37 (13.70%) |  |
| every day or every night | 2 (0.11%) | 10 (1.84%) | 16 (5.93%) |  |
| Night urinate, time |  |  |  | <0.001 |
| 0 | 713 (38.15%) | 64 (11.76%) | 19 (7.04%) |  |
| 1 | 748 (40.02%) | 168 (30.88%) | 39 (14.44%) |  |
| 2 | 309 (16.53%) | 179 (32.90%) | 77 (28.52%) |  |
| 3 | 84 (4.49%) | 87 (15.99%) | 73 (27.04%) |  |
| 4 | 11 (0.59%) | 32 (5.88%) | 26 (9.63%) |  |
| 5 or more | 4 (0.21%) | 14 (2.57%) | 36 (13.33%) |  |
| Total PSA | 1.34 ± 1.25 | 2.01 ± 2.17 | 5.06 ± 8.76 | <0.001 |
| Trouble with urinate |  |  |  | <0.001 |
| no | 1814 (97.06%) | 446 (81.99%) | 180 (66.67%) |  |
| yes | 55 (2.94%) | 98 (18.01%) | 90 (33.33%) |  |
| feeling empty of bladder after urinate |  |  |  | <0.001 |
| no | 104 (5.56%) | 104 (19.12%) | 77 (28.52%) |  |
| yes | 1765 (94.44%) | 440 (80.88%) | 193 (71.48%) |  |

BUN blood urea nitrogen, SBP systolic blood pressure, SUI stress urinary incontinence, UUI Urge urinary incontinence, Total PSA prostate-specific antigen.

The p-value was calculated by the Kruskal-Wallis test for continuous variables and the chi-square test for categorical variables.

Table S4. Baseline characteristics of the women participants classified by UAA grades.

| Characteristics | Total | Gender | | p-value |
| --- | --- | --- | --- | --- |
|  |  | Men | women |  |
| N, count | 8992 | 2680 | 1396 |  |
| Age, year | 59.29 ± 12.04 | 59.08 ± 12.23 | 63.33 ± 11.99 | <0.001 |
| Race, n (%) |  |  |  | <0.001 |
| mexican American | 1316 (14.64%) | 367 (13.69%) | 158 (11.32%) |  |
| other Hispanic | 980 (10.90%) | 249 (9.29%) | 123 (8.81%) |  |
| non-Hispanic White | 4092 (45.51%) | 1090 (40.67%) | 536 (38.40%) |  |
| non-Hispanic Black | 1648 (18.33%) | 705 (26.31%) | 493 (35.32%) |  |
| other Race | 956 (10.63%) | 269 (10.04%) | 86 (6.16%) |  |
| Education, n (%) |  |  |  | <0.001 |
| less Than 9th Grade | 957 (10.65%) | 303 (11.32%) | 179 (12.83%) |  |
| 9-11th Grade | 1064 (11.85%) | 406 (15.17%) | 244 (17.49%) |  |
| high School Grad/GED or Equivalent | 2062 (22.96%) | 657 (24.55%) | 359 (25.73%) |  |
| some College or AA degree | 2671 (29.74%) | 844 (31.54%) | 443 (31.76%) |  |
| college Graduate or above | 2228 (24.81%) | 466 (17.41%) | 170 (12.19%) |  |
| Poverty income ratio | 2.72 ± 1.63 | 2.51 ± 1.59 | 1.99 ± 1.43 | <0.001 |
| Drinking |  |  |  | 0.55 |
| No | 3113 (34.64%) | 955 (35.67%) | 496 (35.53%) |  |
| Yes | 5873 (65.36%) | 1722 (64.33%) | 900 (64.47%) |  |
| Smoking |  |  |  | <0.001 |
| never | 5671 (63.10%) | 1638 (61.17%) | 805 (57.71%) |  |
| current | 1384 (15.40%) | 384 (14.34%) | 234 (16.77%) |  |
| quit | 1933 (21.51%) | 656 (24.50%) | 356 (25.52%) |  |
| BMI | 28.93 ± 6.59 | 32.66 ± 7.95 | 34.28 ± 9.43 | <0.001 |
| MET | 3080.24 ± 4989.61 | 3150.78 ± 5156.27 | 3433.24 ± 5277.44 | 0.193 |
| BUN, mg/dL | 12.80 ± 4.17 | 15.76 ± 5.18 | 23.21 ± 10.93 | <0.001 |
| Serum creatinine, mg/dL | 0.76 ± 0.16 | 0.84 ± 0.23 | 1.25 ± 0.94 | <0.001 |
| Serum uric acid | 4.65 ± 1.08 | 5.62 ± 1.27 | 6.68 ± 1.73 | <0.001 |
| SBP, mmHg | 122.60 ± 16.60 | 136.21 ± 19.37 | 148.83 ± 26.32 | <0.001 |
| Renal failure , n (%) |  |  |  | <0.001 |
| no | 8875 (98.70%) | 2553 (95.26%) | 1110 (79.51%) |  |
| yes but no dialysis | 117 (1.30%) | 123 (4.59%) | 252 (18.05%) |  |
| yes and dialysis | 0 (0.00%) | 4 (0.15%) | 34 (2.44%) |  |
| SUI frequency |  |  |  | <0.001 |
| no | 5025 (55.88%) | 1173 (43.77%) | 593 (42.48%) |  |
| less than once a month | 1941 (21.59%) | 519 (19.37%) | 177 (12.68%) |  |
| a few times a month | 1251 (13.91%) | 465 (17.35%) | 228 (16.33%) |  |
| a few times a week | 496 (5.52%) | 280 (10.45%) | 178 (12.75%) |  |
| every day or every night | 279 (3.10%) | 243 (9.07%) | 220 (15.76%) |  |
| UUI frequency |  |  |  | <0.001 |
| no | 6382 (70.97%) | 1297 (48.40%) | 490 (35.10%) |  |
| less than once a month | 1283 (14.27%) | 391 (14.59%) | 163 (11.68%) |  |
| a few times a month | 891 (9.91%) | 492 (18.36%) | 274 (19.63%) |  |
| a few times a week | 312 (3.47%) | 286 (10.67%) | 238 (17.05%) |  |
| every day or every night | 124 (1.38%) | 214 (7.99%) | 231 (16.55%) |  |
| Night urinate, time |  |  |  | <0.001 |
| 0 | 2438 (27.11%) | 367 (13.69%) | 108 (7.74%) |  |
| 1 | 3708 (41.24%) | 830 (30.97%) | 324 (23.21%) |  |
| 2 | 1856 (20.64%) | 747 (27.87%) | 349 (25.00%) |  |
| 3 | 738 (8.21%) | 454 (16.94%) | 321 (22.99%) |  |
| 4 | 169 (1.88%) | 173 (6.46%) | 159 (11.39%) |  |
| 5 or more | 83 (0.92%) | 109 (4.07%) | 135 (9.67%) |  |

BUN blood urea nitrogen, SBP systolic blood pressure, SUI stress urinary incontinence, UUI Urge urinary incontinence.

The p-value was calculated by the Kruskal-Wallis test for continuous variables and the chi-square test for categorical variables.

Table S5. The correlation between age and urological indicator among the WCNPCS participants.

| Urological indicator | Men | Women |
| --- | --- | --- |
| UUI | 0.0657 | 0.0563 |
| SUI | -0.0037 | -0.0004 |
| Night urinate, time | 0.3839 | 0.2872 |
| SBP | 0.3178 | 0.1033 |
| BUN | 0.057 | 0.1385 |
| Serum creatinine | 0.0195 | 0.0683 |
| Serum uric acid | -0.076 | 0.1243 |
| feeling empty of bladder after urinate | -0.1773 | — |
| Trouble with urinate | 0.1811 | — |

BUN blood urea nitrogen, SBP systolic blood pressure, SUI stress urinary incontinence, UUI Urge urinary incontinence.

Table S6. Baseline characteristics of the study participants from WCNPCS classified by sex.

| Characteristics | Total | Sex | | p-value |
| --- | --- | --- | --- | --- |
|  |  | Men | Women |  |
| N | 656 | 412 | 244 |  |
| Age | 55.47 ± 10.07 | 56.42 ± 9.58 | 53.89 ± 10.67 | <0.001 |
| Education |  |  |  | 0.413 |
| Primary school and below | 344 (52.44%) | 221 (53.64%) | 123 (50.41%) |  |
| Junior high school | 198 (30.18%) | 126 (30.58%) | 72 (29.51%) |  |
| High school and vocational high school | 56 (8.54%) | 33 (8.01%) | 23 (9.43%) |  |
| College diploma and undergraduate | 56 (8.54%) | 30 (7.28%) | 26 (10.66%) |  |
| Graduate and above | 2 (0.30%) | 2 (0.49%) | 0 (0.00%) |  |
| Marriage status | |  |  | <0.001 |
| Married | 585 (89.18%) | 375 (91.02%) | 210 (86.07%) |  |
| Unmarried | 16 (2.44%) | 15 (3.64%) | 1 (0.41%) |  |
| Divorced/Separated | 20 (3.05%) | 9 (2.18%) | 11 (4.51%) |  |
| Widowed | 35 (5.34%) | 13 (3.16%) | 22 (9.02%) |  |
| Smoking |  |  |  | <0.001 |
| Currently | 164 (25.04%) | 156 (37.96%) | 8 (3.28%) |  |
| Occasionally | 10 (1.53%) | 8 (1.95%) | 2 (0.82%) |  |
| Never | 418 (63.82%) | 187 (45.50%) | 231 (94.67%) |  |
| Quit | 63 (9.62%) | 60 (14.60%) | 3 (1.23%) |  |
| Drinking |  |  |  | <0.001 |
| Yes | 256 (39.08%) | 212 (51.58%) | 44 (18.03%) |  |
| No | 399 (60.92%) | 199 (48.42%) | 200 (81.97%) |  |
| Physical activity |  |  |  | 0.807 |
| 1 | 8 (2.21%) | 4 (1.87%) | 4 (2.70%) |  |
| 2 | 43 (11.88%) | 23 (10.75%) | 20 (13.51%) |  |
| 3 | 47 (12.98%) | 28 (13.08%) | 19 (12.84%) |  |
| 4 | 264 (72.93%) | 159 (74.30%) | 105 (70.95%) |  |
| BMI | 25.44 ± 3.72 | 25.41 ± 3.77 | 25.48 ± 3.65 | 0.766 |
| UUI |  |  |  | <0.001 |
| no | 600 (91.46%) | 402 (97.57%) | 198 (81.15%) |  |
| yes | 56 (8.54%) | 10 (2.43%) | 46 (18.85%) |  |
| SUI |  |  |  | <0.001 |
| no | 500 (76.22%) | 409 (99.27%) | 91 (37.30%) |  |
| yes | 156 (23.78%) | 3 (0.73%) | 153 (62.70%) |  |
| feeling empty of bladder after urinate | | |  | 0.587 |
| no | 53 (12.80%) | 53 (12.86%) | 0 (0.00%) |  |
| yes | 361 (87.20%) | 359 (87.14%) | 2 (100.00%) |  |
| Trouble with urinate | |  |  | 0.143 |
| no | 374 (83.30%) | 340 (82.52%) | 34 (91.89%) |  |
| yes | 75 (16.70%) | 72 (17.48%) | 3 (8.11%) |  |
| Night urinate, time | 1.11 ± 1.07 | 1.07 ± 1.06 | 1.18 ± 1.09 | 0.189 |
| Serum creatinine | 0.82 ± 0.28 | 0.90 ± 0.30 | 0.68 ± 0.14 | <0.001 |
| Serum uric acid | 6.48 ± 1.67 | 7.03 ± 1.64 | 5.55 ± 1.26 | <0.001 |
| Urological age | 55.44 ± 10.35 | 56.37 ± 10.09 | 53.86 ± 10.62 | 0.002 |
| UAA | -0.04 ± 3.00 | -0.04 ± 2.84 | -0.03 ± 3.25 | 0.97 |

BUN blood urea nitrogen, SBP systolic blood pressure, SUI stress urinary incontinence, UUI Urge urinary incontinence.

The p-value was calculated by the Kruskal-Wallis test for continuous variables and the chi-square test for categorical variables.

Table S7. Association between UAA and health outcomes in WCNPCS classified by sex

| Health outcomes | Men | Women |
| --- | --- | --- |
|  | OR (95%CI) | |
| Depression | **1.36 (1.11, 1.67)** | **1.22 (1.05, 1.40)** |
| Diabetes | **1.24 (1.05, 1.47)** | 1.05 (0.89, 1.24) |
| CVD | **1.29 (1.01, 1.65)** | **1.30 (1.03, 1.63)** |
|  | β (95%CI) | |
| PHQ score | **0.20 (0.08, 0.33)** | **0.24 (0.03, 0.45)** |
| SWLS | -0.08 (-0.30, 0.14) | **-0.25 (-0.44, -0.05)** |
| IADL | -0.04 (-0.09, 0.02) | -0.03 (-0.11, 0.06) |
| ADL | 0.00 (-0.02, 0.02) | 0.01 (-0.05, 0.06) |
| CMI | -0.00 (-0.10, 0.09) | -0.04 (-0.24, 0.16) |
| NLR | 0.01 (-0.03, 0.05) | -0.01 (-0.05, 0.04) |
| SII | **7.80 (0.31, 15.28)** | -2.01 (-12.33, 8.32) |
| Grip strength | -0.88 (-8.85, 7.09) | -0.08 (-0.29, 0.13) |

PHQ-9 is the Patient Health Questionnaire. CVD are the cardiovascular diseases. SWLS is the Scale to Measure Satisfaction with Life. CVD are the cardiovascular diseases. ADLs are difficulties in activities of daily living. IADLs are difficulties in instrumental activities of daily living. CMI is the Cardiometabolic index. NLR is the neutrophil-to-lymphocyte ratio. SII is the systemic immune-inflammation index.

Figure 1S. Calculate the equation for urological age.

*U*$A=\frac{\sum_{i=1}^{n} \left( x_{i}-q_{i} \right)\frac{k_{i}}{s_{i}^{2}}+\frac{CA}{s_{KD}^{2}}}{\sum_{i=1}^{n} (\frac{k_{i}}{s_{i}})^{2}+\frac{1}{s_{KD}^{2}}}$

*U*$AA=UA-CA$

x is the measured value of the biomarker. For each biomarker i, the parameters k, q, and s are derived from regression estimates of the calendar age of the biomarkers in the reference sample. k, q, and s are the regression intercept, slope, and root mean square error, respectively. The sKD is a scaling factor equal to the square root of the variance of calendar age explained by the biomarker set in the reference sample. CA is the chronological age.

Figure 2S. Association between UAA grades and random blood glucose, CONUT and klotho.


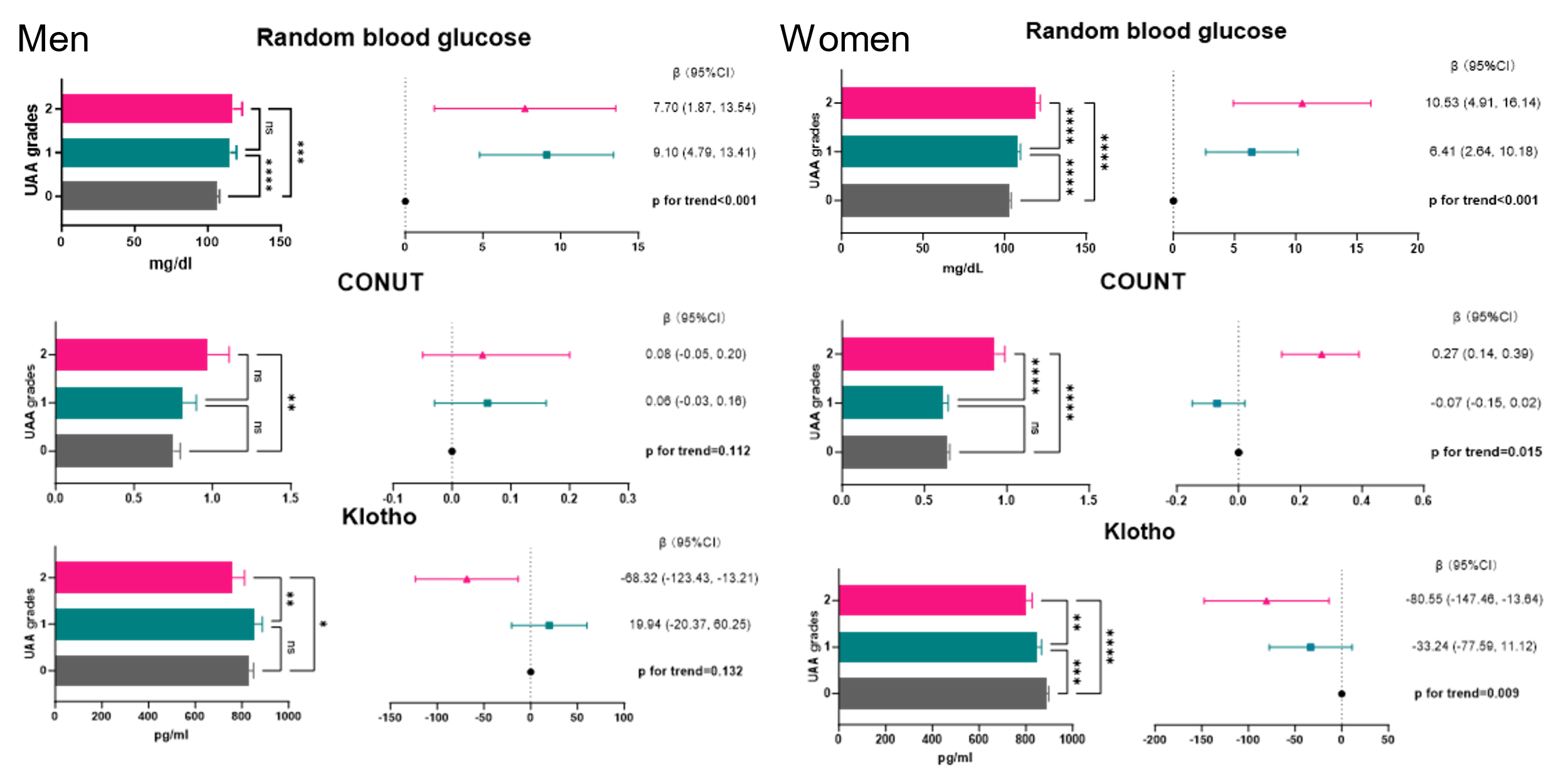


The association adjusted for age, race, education, poverty income ratio, BMI, drinking, smoking, met. CONUT (CONtrolling NUTritional status), UAA (urological age acceleration), CI (confidence interval).

Figure 3S. Association between survival and health outcome with UA/UAA among women under 40 years old.


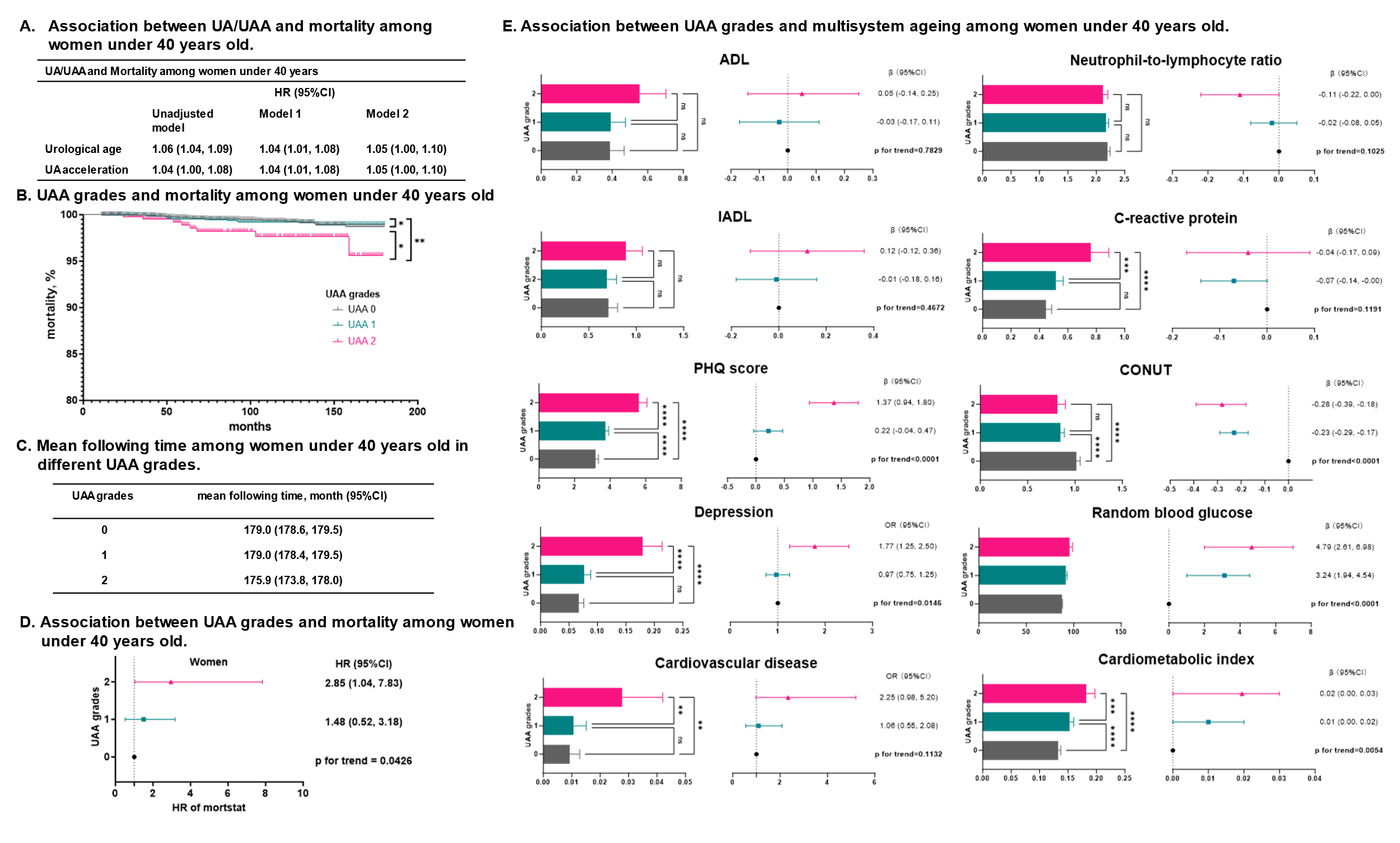


A. Association between UA/UAA and mortality among women under 40 years old. Model 1: adjusted for age, rath, education and poverty income ratio. Model 2 adjusted for age, race, education, poverty income ratio, BMI, drinking and smoking.

B. UAA grades and mortality among women under 40 years old.

C. Mean following time among women under 40 years old in different UAA grades.

D. Association between UAA grades and mortality among women under 40 years old. D. Association between UAA grades and multisystem ageing among women under 40 years old. Model adjusted for age, race, education, poverty income ratio, BMI, drinking and smoking.

E. Association between UAA grades and multisystem ageing among women under 40 years old. Model adjusted for age, race, education, poverty income ratio, BMI, drinking and smoking. Activities of daily living (ADL), instrumental activities of daily living (IADL), The Patient Health Questionnaire (PHQ) score, Cardiometabolic index (CMI), and CONtrolling NUTritional status (CONUT).

Figure 4S. The correlation between each urological indicator in WCNPCS


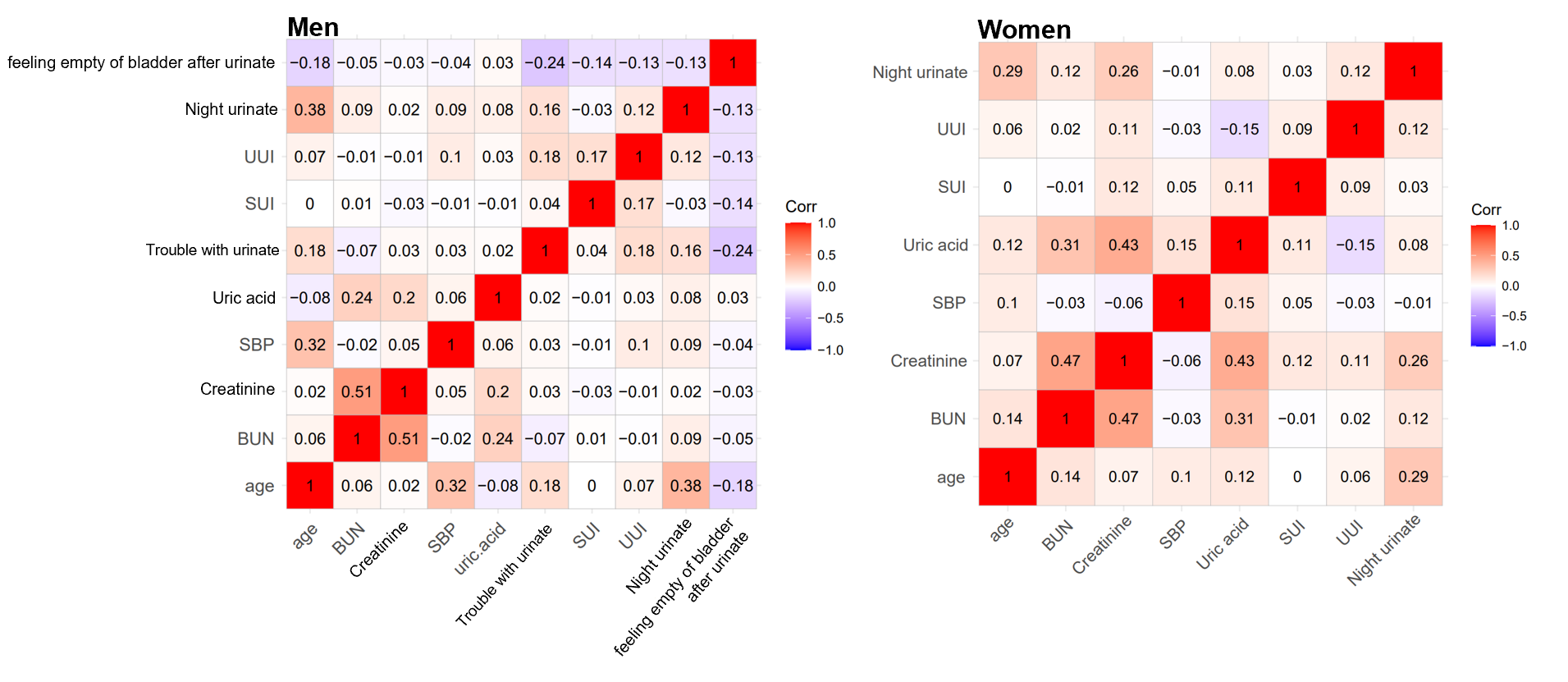


SBP is systolic blood pressure. BUN is blood urea nitrogen. UUI is urge urinary incontinence. PSA is the prostate-specific antigen.SUI is stress urinary incontinence.

Reference:

1. Kenis C, Decoster L, Van Puyvelde K, De Grève J, Conings G, Milisen K, et al. Performance of two geriatric screening tools in older patients with cancer. J Clin Oncol. 2014;32(1):19-26.

2. Spitzer RL, Kroenke K, Williams JB. Validation and utility of a self-report version of PRIME-MD: the PHQ primary care study. Primary Care Evaluation of Mental Disorders. Patient Health Questionnaire. Jama. 1999;282(18):1737-44.

3. Uher R, Payne JL, Pavlova B, Perlis RH. Major depressive disorder in DSM-5: implications for clinical practice and research of changes from DSM-IV. Depress Anxiety. 2014;31(6):459-71.

4. Wakabayashi I, Daimon T. The "cardiometabolic index" as a new marker determined by adiposity and blood lipids for discrimination of diabetes mellitus. Clinica chimica acta; international journal of clinical chemistry. 2015;438:274-8.

5. Jhuang YH, Kao TW, Peng TC, Chen WL, Li YW, Chang PK, et al. Neutrophil to lymphocyte ratio as predictor for incident hypertension: a 9-year cohort study in Taiwan. Hypertension research : official journal of the Japanese Society of Hypertension. 2019;42(8):1209-14.

6. Ignacio de Ulíbarri J, González-Madroño A, de Villar NG, González P, González B, Mancha A, et al. CONUT: a tool for controlling nutritional status. First validation in a hospital population. Nutricion hospitalaria. 2005;20(1):38-45.

7. Torres PU, Prié D, Molina-Blétry V, Beck L, Silve C, Friedlander G. Klotho: an antiaging protein involved in mineral and vitamin D metabolism. Kidney international. 2007;71(8):730-7.

8. Sabanayagam C, Shankar A. Income is a stronger predictor of mortality than education in a national sample of US adults. Journal of health, population, and nutrition. 2012;30(1):82-6.

9. Yu YC, Paragomi P, Wang R, Jin A, Schoen RE, Sheng LT, et al. Composite dietary antioxidant index and the risk of colorectal cancer: Findings from the Singapore Chinese Health Study. Int J Cancer. 2022;150(10):1599-608.

10. Cavicchia PP, Steck SE, Hurley TG, Hussey JR, Ma Y, Ockene IS, et al. A new dietary inflammatory index predicts interval changes in serum high-sensitivity C-reactive protein. J Nutr. 2009;139(12):2365-72.

11. Krebs-Smith SM, Pannucci TE, Subar AF, Kirkpatrick SI, Lerman JL, Tooze JA, et al. Update of the Healthy Eating Index: HEI-2015. J Acad Nutr Diet. 2018;118(9):1591-602.

12. Lloyd-Jones DM, Allen NB, Anderson CAM, Black T, Brewer LC, Foraker RE, et al. Life's Essential 8: Updating and Enhancing the American Heart Association's Construct of Cardiovascular Health: A Presidential Advisory From the American Heart Association. Circulation. 2022;146(5):e18-e43.

13. Golmohammadi M, Ayremlou P, Zarrin R. Higher oxidative balance score is associated with better glycemic control among Iranian adults with type-2 diabetes. Int J Vitam Nutr Res. 2021;91(1-2):31-9.

14. Diener E, Emmons RA, Larsen RJ, Griffin S. The Satisfaction With Life Scale. J Pers Assess. 1985;49(1):71-5.
